# Impact of scaling on aeration performance of fine-pore membrane diffusers based on a pilot-scale study

**DOI:** 10.1038/s41598-020-61814-5

**Published:** 2020-03-17

**Authors:** Mingyue Wang, Huijun Mo, Guo-hua Liu, Lu Qi, Yu Yu, Haitao Fan, Xianglong Xu, Tao Luo, Yuting Shao, Hongchen Wang

**Affiliations:** 1 Low-carbon Water Environment Technology Research Center, School of Environment & Natural Resources, Renmin University of China, Beijing, 100872 China; 2 Shine Water Limited Company, Beijing, 100097 China

**Keywords:** Environmental sciences, Engineering, Materials science

## Abstract

Aeration systems consume a large amount of energy in wastewater treatment plants. Fine-pore membrane diffusers are most commonly used in aeration systems. Scaling and fouling on these membrane diffusers will lead to decreased performance in aeration and increase energy consumption. This pilot-scale study focused on the scaling of the three kinds of fine-pore membrane diffusers under different influent hardness conditions. The results showed that the diffusers were mainly polluted by calcium carbonate scaling. Scaling occurred on the outer surface, orifices and inner surface of the membranes. The dynamic wet pressure (DWP) of ethylene-propylenediene monomer (EPDM), silicone and polyurethane (PU) membrane diffusers increased by 126%, 34% and 304%, respectively, within 50 days when the hardness was 400 mg/L (as CaCO_3_). However, the increase ratio became obviously slow during the subsequent 60-day operation, indicating a scaling rule of membrane diffusers. Considering that the standard aeration efficiency (SAE) acted as a comprehensive index for judging the aeration performance, the silicone diffuser had better performance than the other two diffusers when severe scaling occurred. This research also provides basic support for the design of membrane diffusers to improve their anti-scaling performance.

## Introduction

Biological processes are the core components in wastewater treatment plants (WWTPs). Supplying air to biomass requires the highest energy use^1^, accounting for more than 50% of the net power demand^2^, which represents a significant fraction of total operating costs, ranging from 50% to 80%^3,4^. Since fine-pore diffusers have better performance in terms of specific energy consumption than coarse bubble diffusers^5^, they are extensively used for supplying oxygen to biomass in municipal WWTPs. Currently, fine-pore diffusers are mainly made of ethylene-propylenediene monomer (EPDM), silicone, and polyurethane (PU). For these types of diffusers, a main issue is organic and inorganic pollution forming during the aeration process^6–12^.

Henze^13^ and Metcalf^14^ reported that diffuser fouling was caused by biological mucus formed on the surface, and scaling was caused by the inorganic precipitation of silica, calcium carbonate and calcium sulfate. Rosso *et al*.^15^ found that deposits of Fe, P and Ca existed in the used membrane by collecting diffuser membranes from wastewater treatment plants for pollutant determination after a period of use. Used membrane diffusers have a higher dynamic wet pressure (DWP) than new diffusers. Due to the increase in the DWP of the diffuser, the pressure provided by the blower cannot meet the requirement of fully releasing air from the diffuser, so a higher-powered blower should be used. Manel *et al*.^16^ analyzed the change in the energy consumption of the diffuser and linked the pollution of the diffuser with the demand for aeration energy. Furthermore, they provided the quantitative prediction of aeration energy intensity and cost and investigated the influence of process conditions as well as microbial activity on the deterioration of diffuser performance^17^.

Scaling plays an important role in the pollution of fine-pore diffusers. In the studies of ceramic diffusers, it was found that iron salts, hardness and fine sand in the mixture were partial causes of diffuser scaling^18,19^. Inorganic elements such as Ca, Mg, S and P could also precipitate on the surface of ceramic diffusers^20,21^. Kim and Boyle^11^ found that when the pollution on the surface of the ceramic diffuser was mainly inorganic, such as calcium carbonate, the DWP of the diffuser increased more than that of the biofilm composed of polysaccharides produced by bacterial cells and microorganisms.

Detailed research on scaling of fine-pore ceramic diffusers has been performed. However, few specialized studies have been carried out on the scaling of fine-pore membrane diffusers until now. Some groundwater-based or coastal WWTPs which use membrane diffusers often suffer from serious inorganic scaling, which are tightly attached to the surface of the membrane diffusers^22^. Compared with biofilm fouling, inorganic scaling is more difficult to remove^23^. Therefore, it is necessary to study inorganic scaling on fine-pore membrane diffusers.

The overall goal of this research was to study the variation in aeration performance of fine-pore membrane diffusers after pollution caused by inorganic scaling. Three types of the most widely used membrane diffuser materials (EPDM, silicone and PU) were evaluated in the study. The diffusers were operated continuously for 50 days in tanks. Furthermore, the pollutant composition was determined, and the influence on aeration performance was explained. Using the analysis of diffuser material properties, the scaling mechanism was interpreted. This research is the first to study the inorganic scaling of membrane diffusers and analyze its impact on aeration performance. The present study provides a basis for a comprehensive understanding of fine-pore membrane diffuser pollution to determine anti-pollution measures.

## Materials and Methods

### Materials

**Figure 1:**
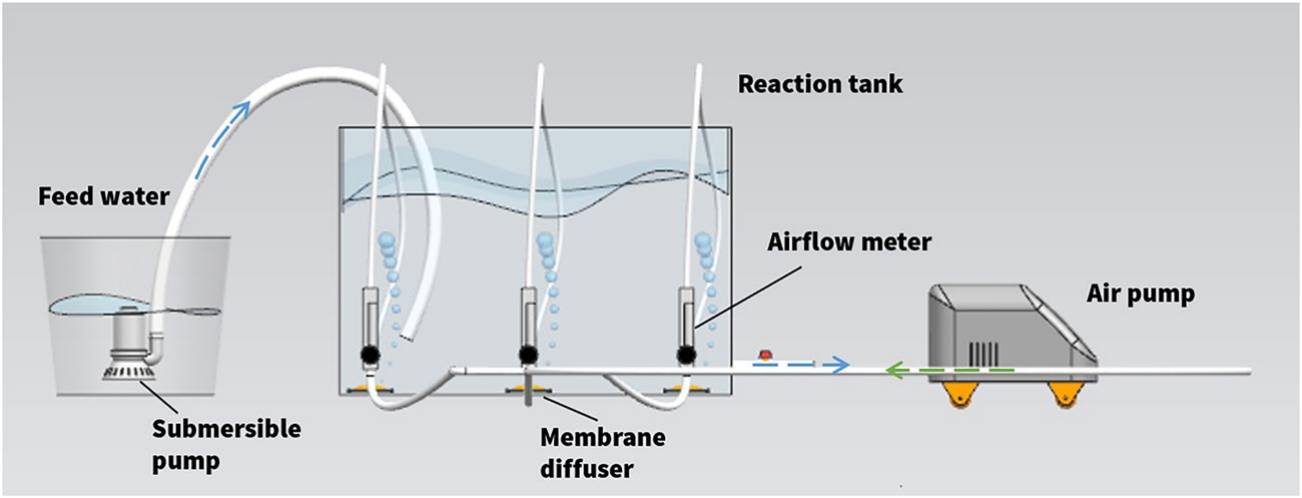
Schematic diagram of the experimental device.

The pilot-scale aeration tank used in this study was made of polymethyl methacrylate and had an effective volume of approximately 180 L, at 800 mm length, 400 mm width and 600 mm high (Fig. 1). Three sets of these devices were used in this experiment.

Three types of fine-pore membrane diffusers (EPDM, silicone and PU) were installed on the bases of the tank, six hundred 1-mm orifices were evenly distributed in each membrane diffuser, and the effective aeration diameter of each diffuser was 85 mm. The thicknesses of the three types of membranes were 2 mm for EPDM, 1.5 mm for silicone and 0.4 mm for polyurethane. Membrane diffusers were supplied by the Environmental Protection Equipment Co., Ltd. (Hebei, China).

Three kinds of feed water with different hardnesses of CaCO_3_ (200 mg/L, 400 mg/L and 800 mg/L) were used in this study. They were diluted tap water (200 mg/L), tap water (400 mg/L), and artificial synthetic water (800 mg/L) with added CaCl_2_ and MgCl_2_ into the tap water, respectively. The feed water was pumped into the tanks daily from the feeding tanks, and the water depth in the tanks was maintained at 0.5 m.

### Methods

#### Experimental procedure

The aeration scaling experiment was operated at 16 ± 2 °C for 50 days. The airflow of the diffusers was 0.3 Nm^3^/h.

To ensure that the orifices on the membrane diffusers were opened, the diffusers were operated at high airflow (0.5 Nm^3^/h) for 3 days before the experiment was started. After 3 days of operation, the membrane diffusers were dismantled from the base and washed to remove the fouling so that the membranes were completely clean when the aeration scaling experiments began operation.

#### Microscopic analysis

Scanning electron microscope (SEM, ZEISS SIGMA, HD) was performed to observe the changes in membrane imaging, including the surface and section. Energy dispersive spectrometer (EDS, Oxford) connecting to the SEM allowed us to identify the chemical element existence in the membranes before and after being scaled, and the thickness of the test area was approximately 5 μm. To ensure the accuracy of the test results, the mean value from four points of each sample was selected for the EDS analysis. X-ray powder diffraction (XRD, Bruker D8 Advance) analysis was employed to identify the crystals of scales scrapped from the surface of the membranes. The surface potential test (Malvern 90) was conducted to identify the surface charging properties of three materials at the initial state and pH 7 of the test solution. The roughness test was conducted to obtain the Ra value of the initial material with a Bruker Contour GT-K. The objective lens was 2.5 × lens, the scanning range was 1.9 × 1.5 mm, and the green light mode was used in the test. Dataphysics DCAT21 was used for the surface energy test, and the two kinds of test solutions were water and glycol.

#### Measurements of diffuser performance

To determine the performance of the diffuser over time, oxygen mass transfer in clean water was measured. The DWP, standard oxygen transfer efficiency (SOTE) and standard aeration efficiency (SAE) were calculated according to the Measurement of Oxygen Mass Transfer in Clean Water for Fine Bubble Diffuser (CJ/T 475-2015)^24^. The DWP was the pressure difference before and after passing through the aerator with a certain ventilation volume (0.3 Nm^3^/h in this study) under 101.325 kPa atmospheric pressure, which was related to the energy consumption. The SOTE was the percentage of oxygen mass transferred to water per unit time in the standard state and test conditions to the oxygen supply of the diffuser. The SAE was the oxygen quality transferred to water by unit useful work consumed by the diffuser under standard and test conditions.

## Results and Discussion

### Aeration performance analysis

**Figure 2:**
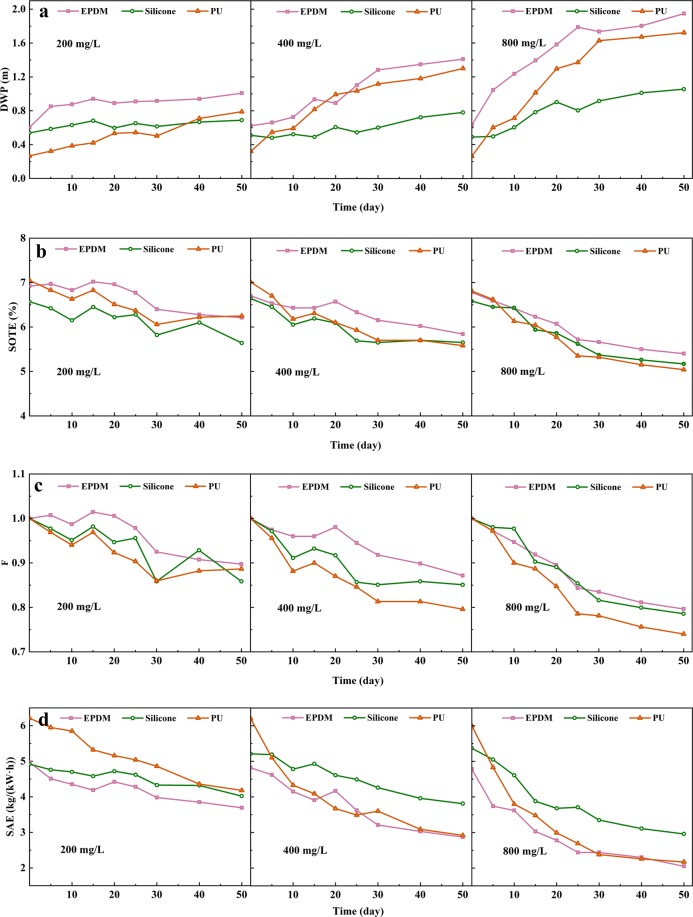
DWP (**a**), SOTE (**b**), Factor F (**c**) and SAE (**d**) of three types of membranes (EPDM, silicone and PU) under different hardness (200, 400 and 800 mg/L, as CaCO_3_) conditions.

The aeration performance assessment for the three membrane diffusers in this study was based on parameters such as DWP, SOTE, Factor F and SAE, and the results are shown in Fig. 2.

#### Dynamic wet pressure

The variations of DWP of the three types of membrane diffusers were assessed under different influent conditions with different hardness. When the hardness was 200 mg/L (as CaCO_3_), the DWP of the three types of membranes increased, but the variety range was small. The DWP of the EPDM membrane diffuser increased from 0.604 m to 1.01 m after 50 days, increasing by 66%. The DWP of the PU membrane diffuser was the smallest at the initial state, at only 0.268 m, but it increased to 0.789 m after 50 days, increasing by 194%, while the silicone diffuser increased from 0.539 m to 0.689 m, only increasing by 28%. For the influent hardness of 400 mg/L (as CaCO_3_), the DWP of EPDM, silicone and PU membrane diffusers increased from 0.623, 0.511 and 0.322 m to 1.41, 0.781 and 1.30 m in 50 days, with an increase ratio of 126%, 34% and 304%, respectively. The results were similar to those of previous studies^11^, which reported that tap water with a hardness of 450 mg/L (as CaCO_3_) led to an increase in DWP for ceramic diffusers from 0.1 m to 0.5 m after operation for 20 days. Meanwhile, the DWP of EPDM, silicone and PU membrane diffusers increased from 0.630, 0.490 and 0.263 m to 1.95, 1.07 and 1.72 m, increasing by 209%, 118% and 554%, respectively, when the hardness was 800 mg/L (as CaCO_3_). The higher the influent hardness, the more the DWP of each membrane increased after the 50-day operation. The EPDM and PU membrane diffusers increased dramatically after the 50-day operation, especially for PU, the DWP of which was five times that of the initial one. The DWP of the silicone membrane diffuser increased the least after 50 days, which was lower than 120%. The slopes of the linear fitting of the DWP data were obtained (Table 1). Compared to the EDPM and PU, the DWP of the silicone diffuser was the most stable. The DWP for the EPDM and PU diffusers increased three or more times faster than that of silicone. Even though the performance of the silicone membrane diffuser was not the best at the beginning, the DWP increased slowly after being polluted and maintained a relatively low DWP.

**Table 1:** Linear fitting results of DWP with time (Slope/R^2^).

Hardness	EPDM	Silicone	PU
200 mg/L	0.00522**/**0.507	0.00218**/**0.453	0.01021**/**0.950
400 mg/L	0.01783**/**0.930	0.00592**/**0.830	0.01914**/**0.892
800 mg/L	0.02382**/**0.807	0.01212**/**0.865	0.03003**/**0.868

#### Oxygen transfer efficiency

As shown in Fig. 2b, when the hardness was 200 mg/L (as CaCO_3_), the SOTE for the EPDM and PU membrane diffusers decreased from approximately 7% to 6.3%, and the silicone diffuser decreased from 6.5% to 5.6%. With an influent hardness of 400 mg/L (as CaCO_3_), the SOTE for the EPDM, silicone and PU membrane diffusers decreased from 6.7%, 6.6% and 7.0% to 5.8%, 5.6% and 5.6%, respectively, after the 50-day operation. The SOTE for the EPDM, silicone and PU membrane diffusers declined from 6.8%, 6.6% and 6.8% to 5.4%, 5.2% and 5.0%, respectively, when the hardness was 800 mg/L (as CaCO_3_). The EPDM diffusers were observed to have better performance in SOTE during the operation under three hardness conditions. The PU membrane diffusers usually have higher SOTE at the beginning of operation but decrease rapidly after being polluted.

The researchers defined the F factor to account for fouling with time in operation^16^:

1 $${\rm{F}}=\frac{SOTE(t)}{SOTE(0)}$$

where SOTE(t) is the standard oxygen transfer efficiency after time t (%), and SOTE(0) is the standard oxygen transfer efficiency at the initial time (%).

The F factor was used here to reflect the variation of SOTE relative to the initial value after scaling. Figure 2c shows that the SOTE for the three fine-pore membrane diffusers decreased under different influent hardness conditions. The higher the hardness was, the smaller the final F value was. When the hardness was 200 mg/L (as CaCO_3_), the F value was 0.88 ± 0.02 after the 50-day operation, while they were all below 0.8 when the hardness was 800 mg/L (as CaCO_3_). The final F values for the EPDM, silicone and PU were 0.796, 0.786 and 0.740, respectively. The SOTE of PU decreased faster than the others, and the EPDM had the highest F value, indicating that the impact of scaling on the SOTE was different for the three types of membrane diffusers, most obviously for the PU, and least obviously for the EPDM. The results were similar to those of previous studies^16^, who reported that after 16 months of operation in an activated sludge system, EPDM and silicone membrane tubes have similar pollution characteristics; the F factor was 0.69 and 0.63, respectively. The final F value of polyurethane was as low as 0.49. The trend of factor F reflected that F values decreased rapidly, mainly in the early stage of operation.

#### Standard aeration efficiency

As shown in Fig. 2d, the SAE showed a downward trend under the three hardness conditions. The higher the hardness was, the faster the SAE decreased. The PU had the best initial SAE. When the hardness was 200 mg/L (as CaCO_3_), the pollution was not serious and had little impact on aeration performance, so PU still maintained the highest SAE after 50 days. However, when the influent hardness increased, the scaling became more serious, and the SAE of PU decreased rapidly at the beginning of operation. In contrast, silicone could have a higher SAE after being polluted. The initial SAE for the EPDM was the lowest, and its pollution effect was relatively great. Therefore, in this study, the SAE of EPDM was always at the lowest level.

Under the high hardness condition, the scaling amount of the membrane was greater than that under the low hardness condition. High hardness conditions can be considered the result of long-term operation of low hardness conditions. It can be inferred that the PU membrane still has the highest SAE at a hardness of 200 mg/L (CaCO_3_) after the 50th day, but with the increase in operation time and the aggravation of pollution, the PU will no longer have an advantage. These results indicate that silicone had a better SAE under high pollution conditions. In the WWTPs, if the hardness is far less than 200 mg/L, the PU can maintain a higher SAE than other materials during long-term operation.

To reveal the continuous change trends in the DWP, the three types of membrane diffusers were operated for 110 days under a hardness of 400 mg/L (as CaCO_3_) condition. The results indicated that the increased ratio of DWP became obviously slow after 70 days of operation (Supplementary Fig. S1). Therefore, the rapid increase in DWP for the three types of membrane diffusers merely occurred at the initial stage of the operation, and it increased slowly afterwards.

Our pilot-scale results revealed that the DWP required by the scaled diffuser increased by one to five times, which caused an increase in the required outlet air pressure of the blower. Therefore, the blowers should be sized to accommodate a twofold or larger increase in DWP over their operation security line^15^. After long-term operation under a hardness of 400 mg/L (as CaCO_3_) condition, it was found that the change rule of the SOTE, Factor F and SAE was similar to DWP (Supplementary Fig. S2–S4), that is, they decreased rapidly in the early stage of operation, then decreased in a gentle way after 70 days of operation. These rules were considered to be more actual. If they have been declining due to scaling pollution, the operation life of the membrane diffusers will be very short.

### Morphology, mass and composition of scales on the membrane

**Figure 3:**
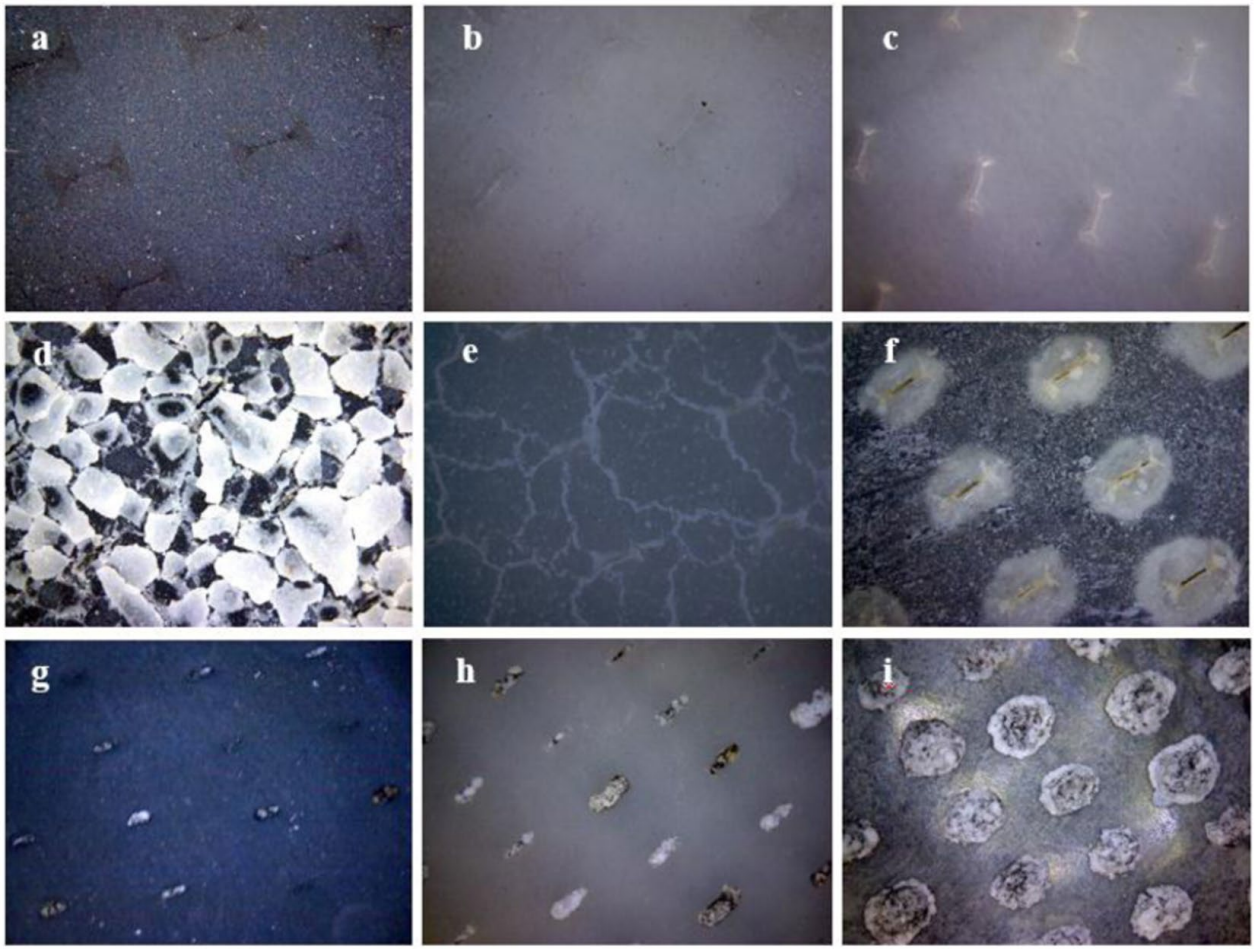
Images taken with hand-held magnifiers of the outer surface of the membranes under a hardness of 800 mg/L (as CaCO_3_) condition. EPDM before (**a**) and after (**d**) scaling, silicone before (**b**) and after (**e**) scaling, PU before (**c**) and after (**f**) scaling, and images of the inner surface of the membranes under a hardness of 800 mg/L (as CaCO_3_) condition for EPDM after scaling (**g**), silicone after scaling (**h**), and PU after scaling (**i**).

The images of the outer surface of the membranes before scaling are shown in Fig. 3a–c. During the 50-day operation, scaling occurred in three membrane diffusers. White scales appeared on the outer surface of the membrane, and the images are shown in Fig. 3d–f. The scales covered the outer surface of the membrane. The scaling morphology on the outer surface of the membrane with different materials was different. The EPDM membrane seems to have flaky scales accumulated on the outer surface, while the silicone and PU membranes have a more uniform distribution of scales. This scaling occurred not only on the surface of the membranes but also in the section and inner surface. The scales accumulated around the orifice even if the inner surface was out of touch with water (Fig. 3g–i and Supplementary Fig. S5d–f). The accumulation of pollutants around the orifice for the PU membrane was the most serious, followed by silicone. This may be related to the thickness of the membrane (EPDM 2 mm, silicone 1.5 mm and PU 0.4 mm). Supplementary Fig. S5a–c shows that before using the membrane, the orifices were clean and closed. This was because the membranes are made of macromolecule material and the orifice is flexible. Compared with the rigid orifices of the ceramic diffuser, this can keep the wastewater from refluxing into the inside of the diffuser when the aeration system stops. However, only 50 days later, many sediments or scales accumulated in the orifices, which blocked and stretched the orifices.

By weighing the mass of the membranes (drying and cooling), the variation of contamination on the membrane was obtained. Δm was introduced:

2 $$\Delta m=\frac{m(t)-m(0)}{m(0)\times S}\times 1000$$

where Δm is the mass unit-area scale (mg/cm^2^), m(t) is the mass of the membrane after time t (g), m(0) is the mass of the membrane at the initial time (g) and S is the effective aeration area of the membrane (cm^2^).

The contamination on the membranes increased linearly during the operation (Supplementary Fig. S6). Compared with other materials, the scales on the PU membrane increased most slowly, and the higher the hardness was, the slower the increase rate was. When the hardness was 800 mg/L (CaCO_3_), the increase in scale on the PU membrane was less than half of that of the other materials. This meant that the PU membrane has the smallest amount of scale. This might be due to the smooth surface of PU and the rough surface of EPDM and silicone (Table 2).

**Table 2:** Zeta potential, roughness and surface energy of three materials.

Material	EPDM	Silicone	PU
Zeta potential (mv)	−12.10	−9.67	−13.40
Roughness (Ra/μm)	4.23	3.94	3.18
Surface energy (SE mJ/m^2^)	29.70	21.37	12.82

As shown in Table 3, the main elements of scale were C, O and Ca. In fact, because the thickness of the EDS test was only 5 μm, the element and content of scale on the membrane could be basically reflected by the analysis of the membrane after scaling. The surface elements of the three membranes were similar after they were polluted, indicating that the scales on the surface of the three materials were similar. The results of EDS analysis showed that the calcium content on the surface was 20.29%, 19.36% and 23.38%, while the magnesium content was 1.83%, 2.37% and 0.85%. This meant that the scales on the membrane were mainly composed of calcium, even if there was an almost equal concentration of calcium and magnesium ions in the water.

**Table 3:** Element weight ratio (%) of the outer surface of the membranes after scaling at a hardness of 800 mg/L (CaCO_3_).

Element	C	O	Ca	Mg	S	other
EPDM	23.46	45.41	20.29	1.83	5.93	3.08
Silicone	21.78	48.04	19.36	2.37	2.85	5.60
PU	19.58	50.34	23.38	0.85	4.72	1.13

**Figure 4:**
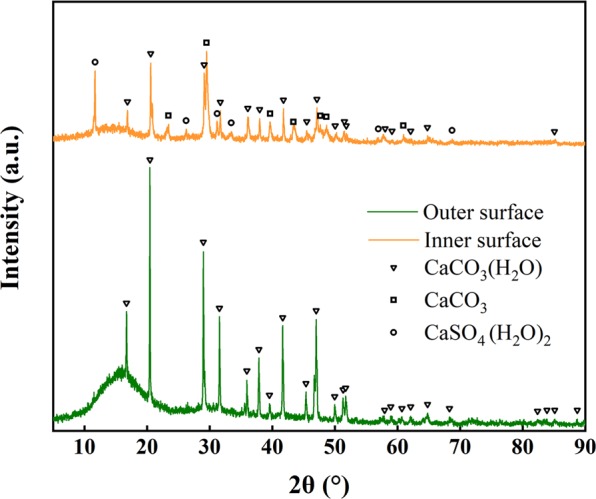
XRD patterns of crystals collected from the PU membrane outer and inner surfaces.

To further understand the composition of the scales, the scale on the membranes of the three materials was scraped off, and XRD analysis was carried out. The XRD pattern of the scale on the outer surface of the PU is shown in Fig. 4. After the analysis with JADE, it was found that there was monohydrocalcite (CaCO_3_ · H_2_O, MHC) crystal in the scale deposit on the outer surface. The same results were obtained by analyzing the scale on the outer surface of EPDM and silicone. This showed that the crystal in the scale on the outer surface was only CaCO_3_ · H_2_O, regardless of the material. However, the crystallinity of the membranes for the different materials was different. The crystallinity of EPDM, silicone and PU was 40.10%, 46.35% and 30.73%, respectively. Meanwhile, the scale on the inner surface of PU was analyzed, and the result is shown in Fig. 4. By comparing the pattern of scale measured with the demo pattern, it can be obtained that the scale on the inner surface of the PU membrane contained monohydrocalcite (CaCO_3_ · H_2_O), calcite (CaCO_3_) and gypsum (CaSO_4_ · (H_2_O)_2_). This indicated that the scale formation on the outer surface and inner surface of PU was not the same. The crystallinity of the scale on the inner surface for the PU was 58.91%, which was higher than that on the outer surface. Through quantitative analysis, the contents of the three kinds of crystalline substances were 60%, and the weight percentage of the three crystals to the total crystal was approximately 60%, 22.4% and 17.6%.

From the analysis results of the EDS and XRD, it can be concluded that the scaling substances on the membrane outer surface were Ca^2+^ deposits. The substance might be a mixture of MHC, amorphous calcium carbonate (ACC) and other Ca deposits. Although the concentration of Mg^2+^ in water was similar to that of Ca^2+^, there was basically no magnesium on the outer surface of the membrane, which was due to the smaller K_sp_ of CaCO_3_ than MgCO_3_.

### Impact of scaling on aeration performance for different fine-pore membrane diffusers

When air passes through the orifice of the diffuser, the DWP consists of the orifice inlet, orifice alongside and orifice outlet loss. The formula of the DWP can be written as follows^25^:

3 $${\rm{DWP}}=\left({{\rm{\xi }}}_{1}+{\rm{\lambda }}\frac{l}{d}+{{\rm{\xi }}}_{2}\right)\frac{{\nu }^{2}}{2g}$$

Due to the small aperture of the diffuser, the airflow in the orifices can be regarded as laminar flow, so Eq. (3) can be written as follows:

4 $${\rm{DWP}}=\left({{\rm{\xi }}}_{1}+\frac{64\upsilon l}{v{d}^{2}}+{{\rm{\xi }}}_{2}\right)\frac{{\nu }^{2}}{2g}$$

where ξ_1_ is the inlet resistance coefficient, ξ_2_ is the outlet resistance coefficient, λ is the alongside resistance coefficient, *l* is the orifice length (m), *d* is the diffuser aperture (m), *v* is the diffuser velocity (m·s ^−1^) and ${\rm{\upsilon }}$ is the kinematic viscosity of air (m^2^·s^−1^).

After the membrane was polluted, on the one hand, the accumulation of pollutants in the orifice caused creep of the membrane, leading to a decrease in ξ_1_ and ξ_2_. On the other hand, the scale on the orifice made ξ_1_ and ξ_2_ larger. In addition, the blockage caused by scaling reduced the number of active orifices and thus increased the gas velocity. This combination led to an increase in DWP (Fig. 2a).

Due to their good strength and tear strength, PUs are usually made into thinner membranes to reduce DWP. Therefore, the DWP was low at the beginning of the operation. However, due to the accumulation of the scales on the inner surface, ξ_1_ and ξ_2_ increase more, and there are more useless orifices resulting in an increase of gas velocity, which made the DWP of PU increase rapidly after the operation. Supplementary Fig. S5d–f shows that the EPDM has the smallest orifice strain, namely, the smallest change in aperture. However, the scaling amount of the EPDM and silicone rubber was similar (Supplementary Fig. S6), indicating that the orifices of the EPDM were seriously blocked relative to the silicone. Therefore, compared with silicone, the increase in gas velocity for the PU membrane was greater, and the DWP increased faster.

One of the main advantages of fine-pore diffusers is the release of smaller bubbles increasing the gas-liquid contact area and improving the oxygen utilization efficiency. Bubble size is the main factor affecting oxygen transfer, thus affecting the overall SOTE. When the membrane is contaminated, it will inevitably affect the release of bubbles. When the membranes started to run, the airflow could make the orifice release bubbles uniformly because the orifice had been opened by high airflow aeration beforehand. In the early stage of operation, scales begin to form on the outer and inner surfaces of the membrane and the orifices. This makes it possible to produce smaller bubbles^26^. Meanwhile, the DWP begins to increase, which leads to a flexible orifice enlargement. This makes the size of the bubbles larger, resulting in a decline in SOTE. With the increase in scaling, some orifices are completely blocked, resulting in disabled bubble release. The airflow remains stable, even if the increase in DWP will make the active orifices larger and the number of active pores decrease; this will lead to the increase in air velocity in each active hole. When the bubbles are formed separately at low gas-flow rates, it is possible to obtain a simple, approximate relationship between the size of the bubble and the size of the orifice^27^:

5 $${\rm{D}}={(d)}^{\frac{1}{3}}{\left(\frac{6\sigma }{\rho g}\right)}^{\frac{1}{3}}$$

where D is bubble diameter (m), d is orifice diameter (m), *σ* is surface tension of the liquid (N/m), *ρ* is liquid density (kg/m^3^) and g is acceleration due to gravity (m/s^2^).

The equation indicates that the diameter of the bubble is independent of the flow rate and increases with the cube root of the orifice diameter. Above a certain flow rate, chain bubbling takes place, and Eq. (5) is no longer valid. Within the range of airflow rates normally encountered in aeration practice, it has been shown that the mean bubble diameter released by fine-pore diffusers, D, is an exponential function of the air flow rate^28^:

6 $${\rm{D}}\propto {G}^{n}$$

where G is the air flow rate (m^3^/s).

As shown in Eq. (6), the diameter of the released bubbles increases because of the increase in flow rate and the larger orifices. Larger bubbles have smaller specific surface areas. Thus, the gas-liquid contact area decreases at a certain airflow, which leads to a decrease in SOTE. At the same time, the increase in gas velocity will lead to an increase in bubble moving speed, and the bubble residence time in water is shortened, which also reduces SOTE.

In terms of material, as mentioned above, the SOTE of EPDM decreases most slowly after scaling, followed by the silicone and PU membranes. The SOTE of PU was significantly affected by scaling. From the XRD analysis of the scales, it can be concluded that the scaling material on the outer surface of the membrane is different from that on the inner surface of the membrane. The crystals on the outer surface of the three materials were all CaCO_3_ · H_2_O, while the crystals on the inner surface of the PU membrane were CaCO_3_ · H_2_O (60%), CaCO_3_ (22.4%) and CaSO_4_ · (H_2_O)_2_ (17.6%). The crystallinity of the scale was also different (40.10% for the EPDM outer surface, 46.35% for the silicone outer surface, 30.73% for the PU outer surface, 58.91% for the PU inner surface). The higher the crystallinity was, the larger the proportion of crystallization area was. The structure of the crystallized zone is denser and harder than that of the non-crystallized zone^29^. This made the blockage tighter and affected the air outlet to a greater extent. Although the PU material had the least amount of scale, its inner surface had an increasingly harder scale, so the blockage was the most serious, and its SOTE decreased more than the other two materials. The EPDM membrane had the smallest creep orifices, so the bubble size was the least different from the initial state, which meant that the SOTE of EPDM was least affected by scaling.

The change in SAE is a comprehensive result of the change in DWP and SOTE. When scaling is light, the PU has a higher SAE, that is, the best aeration performance; when scaling is heavy, silicone is the better choice. Because the influence of scaling on DWP was greater than that on SOTE, the change in DWP was considered to be the main factor influencing SAE, and the change in SOTE was the secondary factor. In the field of material development of anti-scaling membrane diffusers, the change in DWP after scaling should be considered first, followed by the change in SOTE.

### Mechanism analysis of scaling for fine-pore membrane diffusers

In the surface potential test of EPDM, silicone and PU, the zeta potentials of the three materials were −12.10 for EPDM, −9.67 for silicone and −13.40 for PU (Table 2). The zeta potential approximates the potential of the electrostatic charge on the surface of a material in a liquid (usually in an aqueous solution). Generally, when the zeta potential is less than 0—meaning that the surface of the material has a static negative charge—the amount of negative charge is much larger than the amount of positive charge. This suggests that the surface of the three materials could adsorb positive ions^30–32^. PU has a smooth surface (Table 2), which may be the main reason for its minimal pollution. A rough surface is more prone to scale than a smooth surface. The main reason is that the rough surface has a certain roughness and uneven voids, which provides favorable conditions for crystal adhesion and the formation of a crystalline layer^33,34^. Generally, solid surface energy is used to characterize the performance (Table 2)^35^. The rougher the surface is, the higher the surface energy is, the better the adhesion performance is, and the easier the crystalline layer is formed. This means that the higher the surface energy is, the easier the scaling phenomenon occurs on the surface of the membrane. The crystallization process begins with nucleation, and there are more nucleation sites on a rough surface. This may be why the crystallinity of EPDM and silicone is higher than that of PU. At the same time, the mixing of bubbles increases the chance of ion collisions^29,36^, thus accelerating the scaling on the membranes. Most studies have shown that calcium carbonate will infiltrate into the membrane and deposit in the orifices to or even block them^34,37,38^. The scale formation on the outer surface of the membrane extends into the orifices and even reaches the inner surface of the membrane (Supplementary Fig. S5g–i). Although the orifice is venting, the shear force is not sufficient to prevent scaling from occurring in the orifice and on the inner surface of the membrane. The crystallinity of the inner surface of the membrane is higher than that of the surface, which may be due to the single air outlet without flow turbulence at the orifice, resulting in better crystallization conditions. The crystals formed on the outer and inner surfaces are therefore also different.

## Conclusions

In the water where Ca^2+^ and Mg^2+^ exist, the fine-pore membrane diffusers were mainly polluted by calcium carbonate scaling. It occurs on the outer surface, orifices and inner surface of the membrane. After scaling, the performance of the diffusers changed significantly, including the increase in DWP and the decrease in SOTE, causing the decline of SAE comprehensively. Considering SAE as a comprehensive index for judging aeration performance, the silicone diffuser had better performance than the other two diffusers when severe scaling occurred. However, when the scaling was light and had little effect on the aeration performance, the PU diffuser possessed relatively better performance. Although the orifice is venting, the rough and negative surface makes it possible to scale. To increase the anti-scaling performance of the membrane, the surface potential and roughness of the membrane material, as well as the optimum thickness of the membrane, should be designed scientifically.

## Supplementary information

Supplementary information.
