# Supplementary material for: Impact of scaling on aeration performance of fine-pore membrane diffusers based on a pilot-scale study
Source: Sci Rep. 2020 Mar 17;10:4902. doi: 10.1038/s41598-020-61814-5 (PMC7078185; doi:10.1038/s41598-020-61814-5)
Supplement: Supplementary file 1 — Supplementary information. [file 41598_2020_61814_MOESM1_ESM.pdf]

Impact of scaling on aeration performance of fine-pore membrane diffusers based on a pilot-scale study

Mingyue Wang[1], Huijun Mo[1], Guo-hua Liu[1*], Lu Qi[1], Yu Yu[1], Haitao Fan[2], Xianglong Xu[1], Tao Luo[1], Yuting Shao[1], Hongchen Wang[1*]

[1] Low-carbon Water Environment Technology Research Center, School of Environment & Natural Resource, Renmin University of China, Beijing 100872, China

[2] Shine Water Limited Company, Beijing 100097, China

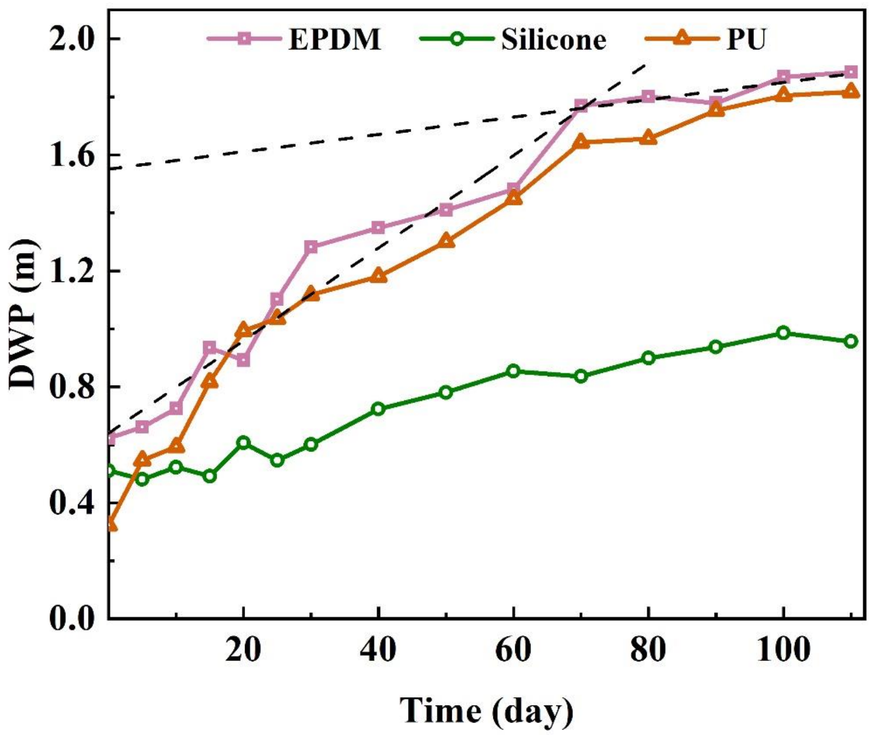

Figure S1. DWP of three membranes (EPDM, silicone and PU) for 110 days with a hardness of 400 mg/L of $CaCO_3$.

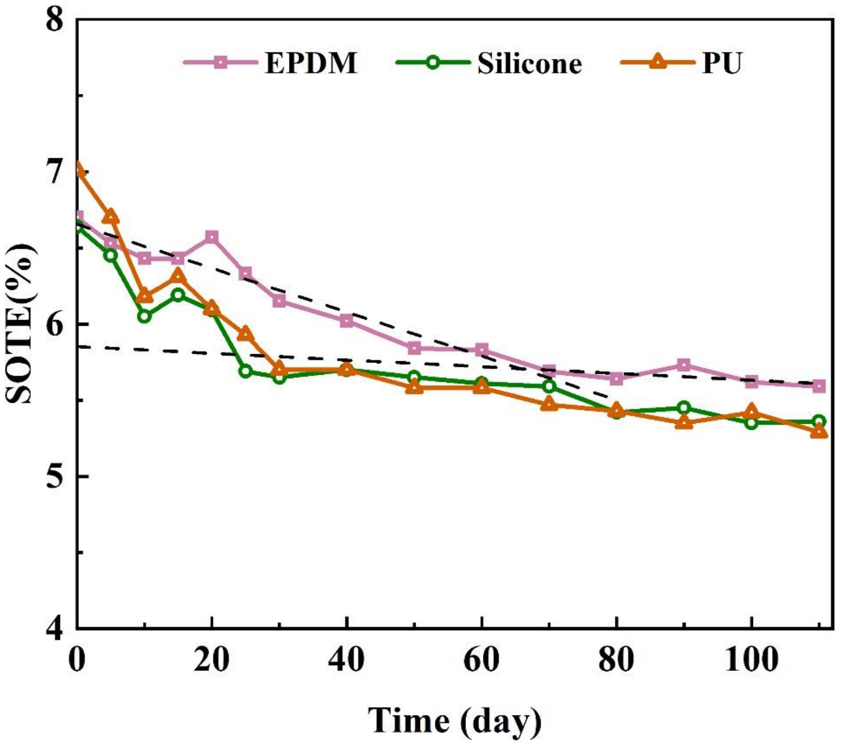

Figure S2. SOTE of three membranes (EPDM, silicone and PU) for 110 days with a hardness of 400 mg/L of $CaCO_3$.

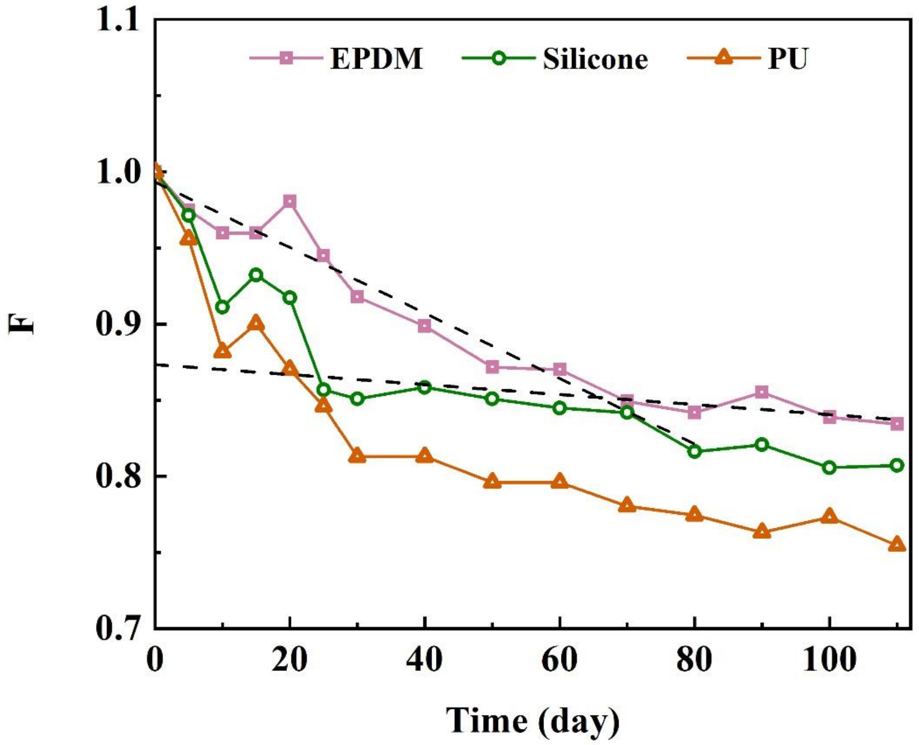

Figure S3. Factor F of three membranes (EPDM, silicone and PU) for 110 days with a hardness of 400 mg/L of $CaCO_3$.

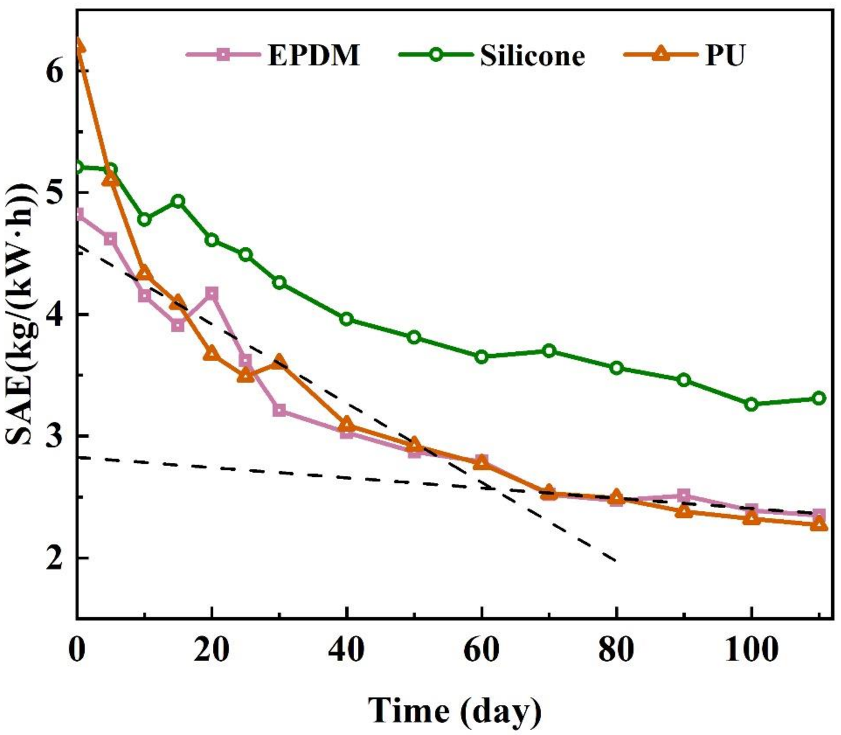

Figure S4. SAE of three membranes (EPDM, silicone and PU) for 110 days with a hardness of 400 mg/L of $CaCO_3$.

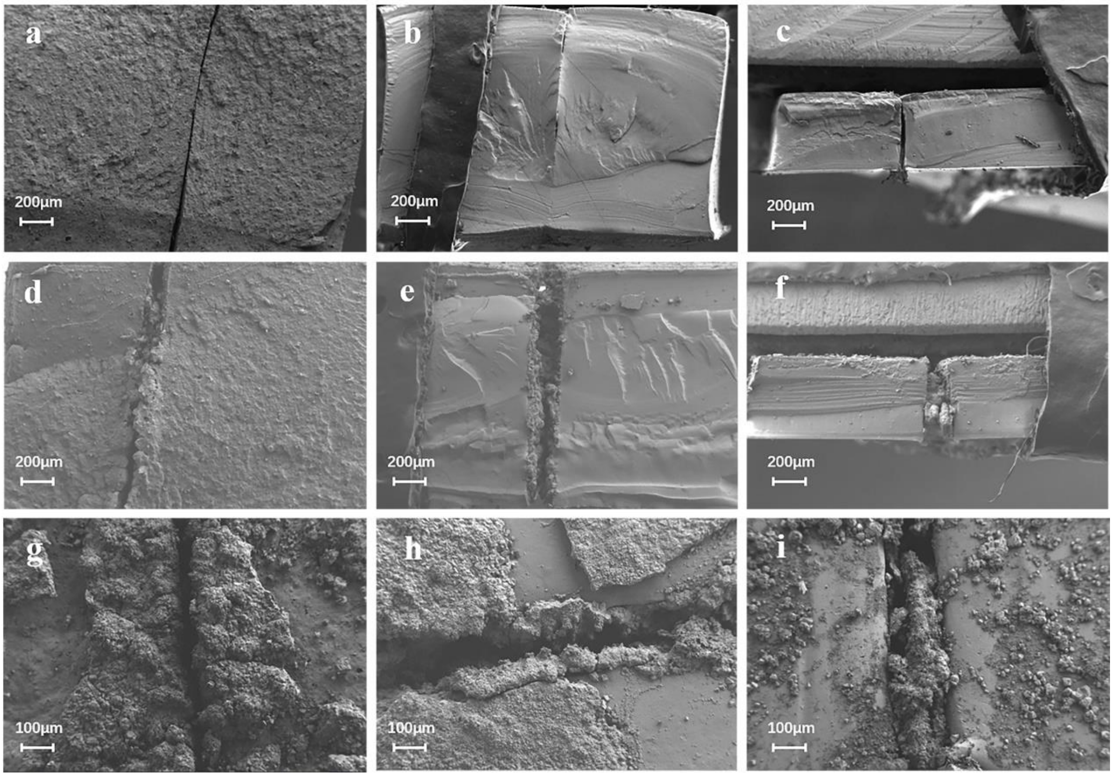

Figure S5. SEM images of section and outer surface of EPDM, silicone and PU membranes after scaling under a hardness of 800 mg/L (as $CaCO_3$) condition. Images of section: EPDM before (a) and after (d) scaling, silicone before (b) and after (e) scaling, PU before (c) and after (f) scaling; and images of outer surface for EPDM after scaling (g), for silicone after scaling (h), for PU after scaling (i).

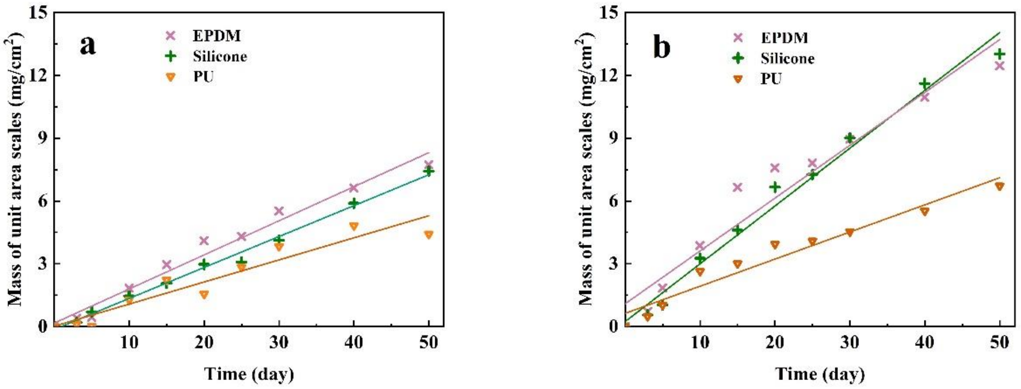

Figure S6. Mass of unit area scales for the three material membranes at different hardness. a: 400 mg/L ($CaCO_3$) ; b: 800 mg/L ($CaCO_3$).
